# Neurodevelopmental Outcome in Very Low Birth Weight Preterm Infants: An Exploratory Multivariable Analysis Including Sonographic Brain Volume Trajectories—Data from the NeoNEVS Project

**DOI:** 10.3390/children13060815

**Published:** 2026-06-13

**Authors:** Simon Loth, Julia Hauer, Marcus Krüger, Renée Lampe, Irina Sidorenko, Alexander Bieber, Christian Brickmann

**Affiliations:** 1 School of Medicine and Health, Department of Pediatrics, Technical University of Munich, TUM University Hospital, 80804 Munich, Germany; 2 Clinic for Neonatology, München Klinik gGmbH, 81545 Munich, Germany; 3 Orthopedic Department, Research Unit for Pediatric Neuroorthopedics and Cerebral Palsy of the Buhl-Strohmaier Foundation, Klinikum Rechts der Isar, School of Medicine and Health, Technical University of Munich, 81675 Munich, Germany

**Keywords:** preterm infant, neurodevelopment, cranial ultrasound, brain volume, machine learning, support vector machine, Bayley scales of infant and toddler development—third edition (Bayley-III), feature importance, interaction analysis

## Abstract

**Highlights:**

**What are the main findings?**

- Respiratory morbidity duration—particularly mechanical ventilation and BPD severity—was the most robustly validated predictor of adverse neurodevelopmental outcome across all Bayley-III domains, surviving stringent Bonferroni correction in univariate analysis and ranking consistently among the top features in the multivariate framework.
- Serial cranial ultrasound-derived brain volume trajectory features contributed conditionally to neurodevelopmental outcome prediction, with their prognostic signal emerging through interactions with gestational maturity rather than as independent univariate associations—a finding only detectable through multivariate interaction analysis.

**What is the implication of the main finding?**

- Machine learning methods are valuable not only as prediction tools but as structured analytical frameworks for characterizing complex predictor–outcome relationships and interactions in small neonatal cohorts, where individual-level prediction remains out of reach.
- Serial bedside cranial ultrasound volumetry represents a scalable, infrastructure-independent approach to longitudinal brain growth monitoring in preterm infants, and its integration with routine clinical variables within a multivariate framework generates specific, testable hypotheses for future prospective validation studies.

**Abstract:**

**Background**: Extremely and very preterm infants are at high risk for adverse neurodevelopmental outcomes. Early prediction remains challenging when relying on static clinical markers or single time-point neuroimaging. Serial cranial ultrasound (CUS) enables repeated bedside assessment of cerebral growth and may provide longitudinal trajectory biomarkers integrable with routine clinical data. **Methods**: In this retrospective two-center cohort study, 89 preterm infants (<32 weeks’ gestation and/or <1500 g birth weight) were assessed using the Bayley-III at 24 months corrected age. Brain volume trajectory features were derived from serial CUS using a standardized ellipsoid model. A three-level analytical framework was applied as follows: univariate regression (62 models, Bonferroni and Benjamini–Hochberg correction), multivariate SVM classification with five-fold GroupKFold cross-validation, ensuring patient-level data separation and feature importance analysis with interaction characterization using stratified Spearman correlation and two-dimensional partial dependence plots. **Results**: Multivariate classification yielded modest but above-chance performance (balanced accuracy 0.277–0.463, Cohen’s κ 0.042–0.152). Respiratory morbidity duration—mechanical ventilation and BPD severity—were the most robustly associated univariate predictors, surviving Bonferroni correction. Brain volume trajectory features showed no significant univariate associations but contributed conditionally within the multivariate framework as follows: the interaction between brain volume slope and trajectory linearity was the strongest for cognitive outcome (Δr = 0.47), and postnatal growth restriction showed amplified adverse effects at lower birth weight for motor outcome (Δr = 0.47). **Conclusions**: This study demonstrates the value of ML methods as structured analytical tools for characterizing predictor–outcome relationships in preterm neurodevelopment; respiratory morbidity and brain volume trajectory features emerged as the most informative predictor classes. Prospective multicenter validation is required before clinical translation.

## 1. Introduction

Preterm birth remains a major global health concern and is associated with substantial neonatal morbidity and long-term neurodevelopmental impairment. Preterm infants (<32 weeks’ gestation) and/or very low birth weight (VLBW) infants (<1500 g) are particularly vulnerable because critical phases of brain growth, connectivity development, and myelination occur during a period of physiologic instability in the neonatal intensive care unit (NICU) [1]. Despite ongoing improvements in survival, impairments in cognition, language, and motor function remain common and create persistent uncertainty for families and clinicians regarding prognosis, follow-up intensity, and the need for early targeted intervention [2,3].

Understanding which perinatal and neonatal factors are most meaningfully associated with later neurodevelopmental outcome—and how these factors interact—is a prerequisite for improving risk stratification and early intervention targeting in this population. Conventional approaches rely on a limited set of static predictors such as gestational age, birth weight, and overt brain injury on imaging. These are clinically informative but provide limited resolution at the individual level, and they do not capture the multifactorial, time-dependent nature of preterm brain development [4,5,6,7]. Risk progression is a dynamic process in which exposures including respiratory instability, neuroinflammation, nutritional deficits, and treatment intensity interact throughout the NICU course to shape developmental trajectories. Prognostic frameworks that incorporate longitudinal, trajectory-based information may therefore provide a richer characterization of predictor–outcome relationships than static approaches alone.

Magnetic resonance imaging (MRI) is the reference standard for neonatal brain assessment, and total cerebral volume at term-equivalent age has been associated with later cognitive and motor outcomes [1,8]. However, MRI is resource-intensive, requires transport outside the NICU, and is rarely feasible for repeated longitudinal assessments in clinically unstable infants [9,10]. Cranial ultrasound (CUS), in contrast, is portable, safe, and routinely performed at the bedside. Standardized linear measurements obtained in coronal and sagittal planes can be combined using an ellipsoid approximation to estimate total brain volume, providing a reproducible surrogate of global cerebral growth [11,12]. Sequential ultrasound-based volumetry captures postnatal brain growth trajectories that correlate with head circumference progression and later neurodevelopmental outcomes [13,14,15,16], and as a longitudinal biomarker integrates the cumulative effects of gestational maturity, respiratory support, inflammation, and nutrition during a critical developmental window [17,18].

Machine learning methods are well-suited to characterizing complex, multivariable predictor–outcome relationships—including non-linear associations and conditional interaction effects—that conventional univariate approaches cannot fully capture [19,20,21,22,23]. Identifying which perinatal and neonatal factors are most meaningfully associated with later neurodevelopmental outcome—and how these factors interact—is a prerequisite for improving risk stratification and targeting early intervention in this vulnerable population. In the context of preterm neurodevelopmental research, where individual risk factors may exert effects that depend substantially on co-occurring exposures or gestational maturity, this capacity for interaction characterization is particularly relevant. Systematic reviews highlight both the promise of ML in this area and its recurring limitations, including small cohort sizes, risk of data leakage in repeated-measure designs, and limited external validation [24,25,26,27]. Neuroimaging-based approaches—including multimodal frameworks combining MRI with clinical data—further extend this capacity, though their applicability in routine practice is limited by infrastructure requirements [28,29,30,31,32,33].

The present study—part of the NeoNEVS Project—aims to characterize the multivariate relationships among serial CUS-derived brain volume trajectory features, routine clinical variables, and neurodevelopmental outcome in very low birth weight preterm infants, with particular focus on identifying conditional predictor interactions that are not detectable by univariate approaches.

## 2. Methods

### 2.1. Study Design, Population and Clinical Assessment

This retrospective cohort study included 89 extremely and very preterm infants treated at two tertiary neonatal intensive care units between 2019 and 2021. Inclusion criteria were: gestational age <32 weeks at birth, birth weight <1500 g, availability of serial CUS examinations from birth to discharge, complete clinical data, and neurodevelopmental assessment at 24 months corrected age using the Bayley-III. Exclusion criteria included major congenital malformations, chromosomal abnormalities, severe brain injury (IVH grade III–IV, cystic PVL), and incomplete follow-up data.

Serial CUS examinations were performed at both centers using the same ultrasound system (Philips Affinity 70; Philips C 8–5 MHz broadband microconvex transducer) by neonatologists trained in neonatal neurosonography. Examinations followed an institutionally standardized schedule consistent with national recommendations as follows: Days of Life 1, 3, 7, 14, and 28, thereafter every 14 days until discharge, with additional scans as clinically indicated. Image acquisition adhered to German Society for Ultrasound in Medicine guidance using predefined coronal and sagittal planes with standardized documentation. Stored images were re-evaluated offline in the institutional PACS; linear brain dimensions were measured directly on archived frames. Observers performing ultrasound examinations were not blinded to clinical variables, as examinations were conducted as part of routine care; however, neurodevelopmental assessments at 24 months were performed by trained developmental specialists blinded to clinical history and ultrasound findings. Inter-center standardization was ensured through use of the same equipment, identical acquisition protocol, and standardized offline measurement procedures at both sites [17]. Total brain volume was estimated from three orthogonal linear measurements (biparietal diameter, fronto-occipital diameter, and cranio-caudal height) using the ellipsoid formula [11]:

Volume = (π/6) × BPD × FOD × CCH

Per-patient trajectory features were derived from the longitudinal measurement series as follows: area under the volume-over-time curve (AUC), slope and linearity (R^2^) of a linear regression against postnatal age, and first and last measured volume. Measurement reproducibility was established previously (intra-observer ICC 0.943–0.980; inter-observer ICC 0.917–0.981 for linear dimensions; 0.975–0.981 for TBV) [17].

### 2.2. Clinical Variables and Neurodevelopmental Assessment

Comprehensive clinical data were collected from electronic medical records, including perinatal variables, anthropometric measurements, respiratory support duration, and neonatal morbidities; full details have been reported previously [17].

Input variables comprised gestational age, sex, delivery mode, Apgar scores, umbilical cord pH, invasive and non-invasive ventilation duration, BPD, PDA, sepsis, NEC/FIP, IVH grades I–II, weight/length/head circumference percentiles at birth and discharge, postnatal anthropometric percentile changes, and the brain volume trajectory features listed above.

Neurodevelopmental outcome was assessed at 24 months corrected age using the Bayley-III across three domains (cognitive, language, and motor). Percentile ranks were used as primary outcome measures; a combined score was calculated as the mean of the three domain-specific percentile ranks. Percentile ranks were selected for their direct normative interpretability; the combined score is a secondary exploratory summary measure. Regression on continuous percentile ranks was additionally tested as an alternative to ordinal classification. Assessments were conducted by trained developmental specialists blinded to clinical history and ultrasound findings. Bayley-III percentile ranks were discretized into the following three ordinal outcome groups: Group 1 (severely impaired, <17th percentile rank), Group 2 (moderately impaired, 17–50th percentile rank), and Group 3 (unimpaired, >50th percentile rank) [34,35,36,37].

### 2.3. Data Processing and Machine Learning:

Data processing and analysis were performed in Python 3.12 using scikit-learn (version 1.8.0), NumPy, pandas, SciPy, and matplotlib. Because each infant contributed multiple serial ultrasound observations, prevention of data leakage between training and evaluation was a critical methodological requirement. A standard random split of observations would result in measurements from the same patient appearing in both the training and test sets, producing inflated performance estimates that cannot generalize to new patients. To address this, 5-fold GroupKFold cross-validation was applied with grouping at the patient level, ensuring that all observations from a given infant were assigned exclusively to either the training or the evaluation fold. This approach reflects the realistic clinical scenario in which the model must generalize to patients not seen during training.

Prior to classification, patient-level feature vectors were constructed by aggregating the longitudinal ultrasound measurements into the trajectory features described above. Missing values were imputed using the median of the training fold to prevent information leakage. Sepsis, as a nominal variable with multiple unordered categories, was one-hot encoded; all other categorical variables were encoded ordinally as clinically appropriate. Class imbalance across the three outcome groups was addressed using balanced class weighting in all applicable models.

The following seven classification algorithms were evaluated: Logistic Regression (L2 regularization), Naive Bayes, k-Nearest Neighbors (k = 5), SVM with linear kernel (SVM-Linear), SVM with radial basis function kernel (SVM-RBF), Random Forest, and Gradient Boosting. The SVM with RBF kernel (C = 1, gamma = ‘scale’) was selected as the primary model based on cross-validated balanced accuracy across all four domains (Supplementary Table S5). All algorithms used scikit-learn default hyperparameters with balanced class weighting; no hyperparameter tuning was performed. Model selection and final performance estimation were performed within the same 5-fold GroupKFold structure; nested cross-validation was not applied. This conflation of model selection and evaluation is a recognized methodological limitation: the reported performance metrics for the selected model (SVM-RBF) may carry optimistic bias of unknown magnitude and should be interpreted accordingly.

Feature importance, directionality, and interaction analyses were conducted using a separate Random Forest classifier trained on the full dataset, independent of the SVM-based classification. This approach was chosen because SVM does not natively provide interpretable feature importance measures; Random Forest MDI and partial dependence plots offer a transparent, model-specific tool for exploratory characterization of predictor–outcome relationships. These analyses are explicitly framed as a separate exploratory layer and do not purport to explain the mechanisms underlying SVM classification performance. Predictor intercorrelations were assessed using Spearman rank correlations across all feature pairs prior to multivariate analysis.

### 2.4. Statistical Analysis

Cross-validated model performance was evaluated using balanced accuracy (mean per-class recall, correcting for class imbalance), F1-score (macro-averaged), Cohen’s κ (quadratic weighted, penalizing larger misclassifications more heavily), MAE and RMSE (in ordinal class units, scale 1–3), and R^2^.

Feature importance was assessed using Mean Decrease in Impurity (MDI) from a Random Forest classifier trained on the full dataset, which quantifies each feature’s contribution to node splitting averaged across all trees. Statistical significance of individual feature contributions was evaluated using permutation-based importance testing (200 permutations per feature), yielding empirical two-sided *p*-values without correction for multiple comparisons; results are therefore interpreted as exploratory. Bootstrap confidence intervals (200 iterations) were derived for MDI estimates. The directionality of each feature’s association with outcome was assessed using Spearman rank correlations between predictor values and Bayley-III group assignments.

To characterize conditional interaction effects—that is, how the predictive association of one feature with outcome depends on the level of a second feature—two-dimensional partial dependence plots (2D PDPs) were computed for the top-ranked feature pairs per domain. An interaction strength metric (Δr) was defined as the absolute difference between stratum-specific Spearman correlations in subgroups split at the median of the stratification variable; a high Δr indicates that the feature–outcome relationship changes substantially depending on the co-variable level.

Univariate predictor–outcome associations were assessed using simple linear regression across all 62 predictor–outcome combinations. To account for multiple comparisons, *p*-values were adjusted using both Bonferroni correction (α = 0.05/62 = 0.00081) and the Benjamini–Hochberg false discovery rate procedure (FDR = 5%). Descriptive statistics are presented as mean ± SD or median (IQR) for continuous variables and as frequencies and percentages for categorical variables.

## 3. Results

### 3.1. Cohort Characteristics

A total of 89 extremely and very preterm infants with complete clinical, sonographic, and neurodevelopmental data were included in the analysis. Baseline and morbidity characteristics are presented in Table 1 and Table 2.

The mean gestational age was 28.3 ± 2.0 weeks (range 23 + 3 to 31 + 6 weeks), and mean birth weight was 1019 ± 285 g (range 395–1490 g). The cohort was predominantly female (60%) and delivered by cesarean section (97%). All but one infant required non-invasive respiratory support; invasive mechanical ventilation was required in 37 infants (42%). The most common neonatal morbidities were sepsis (33%), patent ductus arteriosus (22%), and bronchopulmonary dysplasia (15%). Severe brain injury (IVH grade III–IV) was an exclusion criterion; mild IVH (grades I–II) was present in 12 infants (13%).

### 3.2. Brain Volume Growth

Brain volume measurements and growth trajectory characteristics are summarized in Table 3. Mean brain volume at first measurement was 158 ± 38 cm^3^ (range 77–243 cm^3^) and increased to 275 ± 56 cm^3^ (range 140–454 cm^3^) at the final examination prior to discharge. The mean daily volumetric brain growth rate was 2.4 ± 0.9 cm^3^/day (range from −0.84 to 5.39 cm^3^/day). A mean of 6.6 ± 2.3 ultrasound examinations per infant were available (range 2–13), providing longitudinal trajectory data across the full NICU course.

### 3.3. Neurodevelopmental Outcomes

Neurodevelopmental outcomes at 24 months corrected age are presented in Table 4. Mean Bayley-III percentile ranks were 72 ± 31 for cognitive development (range 1–100), 55 ± 33 for language (range 1–98), 67 ± 25 for motor development (range 2–99), and 65 ± 26 for the combined score (range 1–99). Substantial variability was observed across all domains, reflecting the heterogeneous developmental trajectories of this high-risk population. Language showed the greatest outcome variability (coefficient of variation 60%), followed by cognitive (43%) and motor (37%) domains. For the multivariate classification analysis, percentile ranks were discretized into the following three outcome groups: Group 1 (severely impaired, ≤16th percentile): cognitive 10%, language 20%, motor 7%, and combined 8%; Group 2 (moderately impaired, 17th–50th percentile): cognitive 17%, language 20%, motor 19%, and combined 20%; Group 3 (unimpaired, >50th percentile): cognitive 73%, language 60%, motor 74%, and combined 72%. The preponderance of infants in Group 3 reflects the exclusion of severe brain injury and is an important determinant of classification difficulty, as discussed below.

### 3.4. Model Performance

#### 3.4.1. Level 1—Univariate Associations

**Figure 1:**
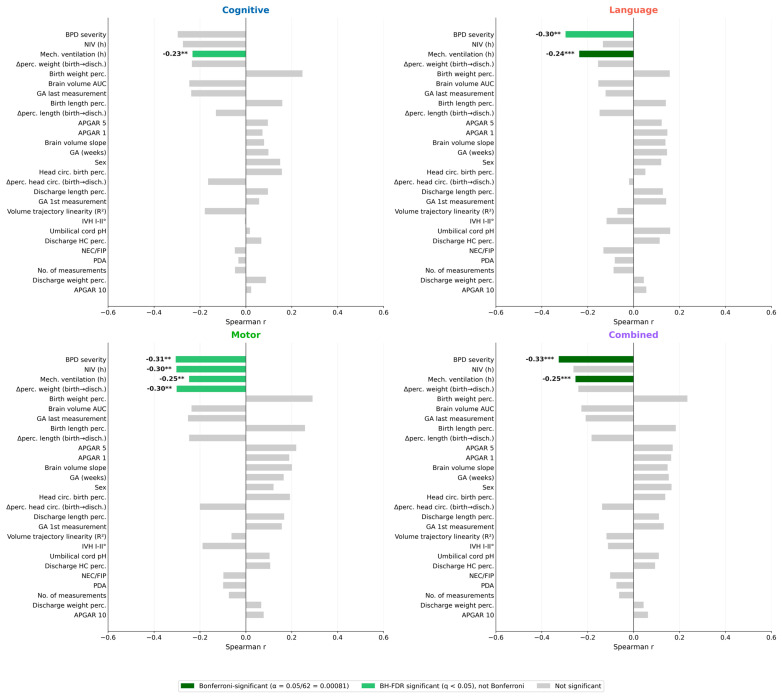
Univariate associations between clinical predictors and Bayley-III outcomes (*n* = 89). Bar length displays the Spearman rank correlation coefficient (r) between each predictor and the continuous Bayley-III percentile rank across four developmental domains (positive r = better outcome; negative r = worse outcome). Features are sorted by mean |r| across all four domains. Bar color reflects the significance of the corresponding simple linear regression model after correction for 62 predictor–outcome combinations: dark green = Bonferroni-significant (α = 0.05/62 = 0.00081); light green = Benjamini–Hochberg FDR significant (q < 0.05), not Bonferroni; gray = not significant. Annotated values show Spearman r with SLR-based significance asterisks for significant associations only (** *p* < 0.01, *** *p* < 0.001). Abr.: r = Spearman rank correlation; BPD = bronchopulmonary dysplasia; NIV = non-invasive ventilation; GA = gestational age; AUC = area under the curve; Δperc. = postnatal percentile change birth to discharge.

As a first analytical level, univariate simple linear regression was performed between each clinical predictor and the continuous Bayley-III percentile rank across all 62 predictor–outcome combinations (Figure 1). This approach serves to identify the individual predictors most robustly associated with outcome and to provide a reference against which the added value of the multivariate analysis can be judged.

After Bonferroni correction (α = 0.05/62 = 0.00081), the following three associations remained significant: mechanical ventilation duration for language (R^2^ = 0.130, *p* = 0.00051) and combined outcome (R^2^ = 0.143, *p* = 0.00026), and BPD severity for combined outcome (R^2^ = 0.125, *p* = 0.00066). After BH-FDR correction (5%), nine associations survived, additionally encompassing mechanical ventilation across all four domains (R^2^ = 0.088–0.143), BPD severity across all four domains (R^2^ = 0.086–0.125), and NIV duration for motor outcome (R^2^ = 0.096). After BH-FDR correction (5%), nine associations survived, additionally encompassing mechanical ventilation across all four domains (R^2^ = 0.088–0.143), BPD severity across all four domains (R^2^ = 0.086–0.125), and NIV duration for motor outcome (R^2^ = 0.096). Spearman rank correlations with the continuous percentile rank confirmed the directionality as follows: BPD (r = −0.30 to −0.33), NIV duration (r = −0.27 to −0.32), mechanical ventilation duration (r = −0.23 to −0.25), and postnatal weight percentile decline (r = −0.23 to −0.29) were consistently and negatively associated with outcome across domains, while birth weight percentile showed a positive association (r = +0.23 to +0.28). Brain growth rate showed no significant univariate association with any Bayley-III domain (all *p* > 0.08). Together, these univariate results establish respiratory morbidity burden as the most robustly identifiable individual predictor class in this dataset.

Several predictors showed substantial intercorrelations, most notably among BPD severity, mechanical ventilation duration, and NIV duration (Spearman r = 0.52–0.62, all *p* < 0.001), and between GA at birth and NIV duration (r = −0.81, *p* < 0.001; Supplementary Figure S1). These collinearities reflect the biological interdependence of respiratory morbidity markers and gestational maturity and suggest that the three respiratory variables should be interpreted as a coherent predictor class rather than independent signals. All variables were retained for the multivariate analysis, as both SVM and Random Forest are robust to correlated features and do not require orthogonal predictors.

#### 3.4.2. Level 2—Multivariate Classification Performance

As a second analytical level, seven classification algorithms were compared using five-fold GroupKFold cross-validation with patient-level grouping, to explore whether combining all predictors simultaneously yields additional discriminative information beyond univariate associations. The SVM with RBF kernel performed most consistently and was selected as the primary model (Table 5).

Balanced accuracy ranged from 0.277 (±0.079) for motor to 0.463 (±0.135) for the combined score. For three domains—cognitive (0.377), language (0.331), and combined (0.463)—performance exceeded the chance level of 0.333 for a three-class problem, indicating modest but non-trivial discriminative capacity. Motor outcome (0.277) fell below chance level, indicating that the classifier did not achieve meaningful discrimination for this domain and should be interpreted as model failure for motor outcome specifically. Cohen’s κ ranged from 0.042 (language) to 0.152 (combined), indicating slight to fair agreement beyond chance. The limited absolute classification performance is consistent with the strong class imbalance as follows: with 60–74% of patients in Group 3, the classifier is confronted with a task in which the majority class dominates, and minority-class signal is sparse. MAE values of 0.640–0.910 ordinal class units indicate that predicted assignments deviated on average by less than one group step from the true outcome. The negative R^2^ values reported in Table 5 arise because R^2^ is here computed between predicted ordinal class assignments (one, two, or three) and observed continuous Bayley-III percentile ranks, measured against a naïve mean-prediction baseline. When coarse three-class assignments do not closely track within-group percentile variation—as expected in any classification problem with broad outcome groups—R^2^ can be negative without implying performance below chance in the categorical sense; balanced accuracy and Cohen’s κ remain the appropriate primary metrics for evaluating classifier quality in this context. Group 2 (moderately impaired) was most frequently misclassified across all domains, reflecting the inherent difficulty of distinguishing an intermediate outcome group from both adjacent classes. These results indicate that multivariate classification yields a modest but consistent signal above chance across all four domains, while falling short of clinically actionable individual-level prediction at this sample size—a finding that is itself methodologically informative, as it helps establish the realistic ceiling of what cross-validated models can contribute with this predictor set and cohort size. Regression on continuous percentile ranks yielded no predictive signal under patient-level cross-validation (SVR and Ridge: R^2^ < 0, Spearman r = 0.009–0.196, all *p* > 0.06), confirming that the limited performance reflects the available predictor set rather than outcome discretization.

#### 3.4.3. Level 3—ML-Based Characterization of Predictor Trajectories, Directionality, and Interactions

As a separate exploratory analytical layer—methodologically independent of the SVM-based classification—a Random Forest classifier was trained on the full dataset to characterize the structure of predictor–outcome relationships. This model was chosen for its native interpretability through MDI feature importance and partial dependence plots; the resulting analyses do not explain the mechanisms of SVM classification but provide a complementary characterization of which predictors matter and under which conditions. MDI feature importance (Supplementary Figures S2–S5) identified NIV duration as a top-ranked predictor across all domains, with negative Spearman correlations confirming that longer ventilation was associated with worse outcome (r = −0.25 to −0.33, *p* < 0.05). Mechanical ventilation showed analogous directionality (r = −0.24 to −0.30). Brain volume trajectory features—including AUC, slope, and trajectory linearity (R^2^)—ranked consistently within the top 10 across all domains. Notably, volume trajectory linearity (R^2^) achieved the highest MDI for motor outcome (0.071) despite a non-significant univariate correlation (r = −0.054, n.s.), indicating a conditional effect requiring multivariate context; permutation testing confirmed significance for motor outcome (*p* = 0.030), NIV for language (*p* = 0.005), and mechanical ventilation for combined outcome (*p* = 0.010).

Interaction analysis (2D PDPs, Supplementary Tables S1–S4) revealed that brain volume slope interacted strongly with GA at first measurement for motor outcome (Δr = 0.41) as follows: slope correlated positively with outcome at low GA (r = +0.43) but not at higher GA (r = +0.02), identifying gestational maturity as a key effect modifier of the brain growth–outcome relationship. Similarly, the adverse association of postnatal weight percentile decline with outcome was amplified in infants with higher GA at last measurement across cognitive, language, and combined domains (Δr = 0.35–0.39). NIV duration showed an analogous interaction with GA at first measurement for language and motor outcomes (Δr = 0.29–0.34).

Full MDI rankings with directionality and permutation test results for all four Bayley-III domains are provided in Supplementary Figures S2–S5; complete feature interaction rankings are provided in Supplementary Tables S1–S4.

## 4. Discussion

*Principal Findings:* This study applies a structured three-level machine learning framework to characterize neurodevelopmental outcome at 24 months corrected age in 89 extremely and very preterm infants, integrating serial CUS-derived brain volume trajectory features with routinely available clinical variables. The primary aim was not individual-level prediction—which, under rigorous patient-level cross-validation, proved not reliably achievable in this cohort—but rather the systematic identification of which predictors matter, in what direction, and under which conditions. A balanced accuracy of 0.277–0.463 and Cohen’s κ of 0.042–0.152 across four Bayley-III domains indicate modest but above-chance discriminative capacity, falling short of robust individual-level classification at this sample size, while the univariate and feature importance analyses reveal a consistent and clinically interpretable predictor–outcome landscape.

*ML as an Analytical Tool: Characterizing Trajectories and Conditional Relationships:* A central premise of this study is that a structured multi-level framework can systematically map the predictor–outcome landscape in a small clinical cohort. The ML methods applied here allow simultaneous screening of all predictor pairs without pre-specification of interaction terms and visualization of conditional relationships through partial dependence plots—advantages that are practical rather than fundamental, as the identified interaction structures could in principle also be approached using conventional regression with pre-specified interaction terms. The transition from asking how well outcomes can be predicted to which variable matter, in what direction, and under which conditions reframes the contribution of a small-cohort ML study from a performance benchmark to a hypothesis-generating clinical characterization [34,35].

The interaction between brain volume slope and volume trajectory linearity for cognitive outcome (Δr = 0.47) illustrates this directly as follows: among infants with irregular growth patterns, brain volume slope was strongly predictive of cognitive outcome, whereas this association was attenuated in infants with consistent trajectories. Similarly, the interaction between postnatal growth restriction and birth weight percentile for motor outcome (Δr = 0.47) demonstrates that the adverse developmental consequences of extrauterine growth restriction are substantially amplified at lower birth weight—a conditional relationship not detectable in any single univariate model [2,4,36]. The interaction between trajectory linearity and brain volume AUC for combined outcome (Δr = 0.44) further indicates that the developmental relevance of cumulative brain volume accumulation depends on the consistency of the growth curve.

*Interpretation of Model Performance:* The limited absolute classification performance is primarily explained by severe class imbalance as follows: with 60–74% of patients in Group 3 (unimpaired), the classifier is confronted with sparse minority-class signal, making balanced accuracy the only appropriate primary metric. This distribution reflects the deliberate exclusion of severe brain injury from the cohort and is therefore a study design feature rather than a data quality issue. Systematic reviews emphasize that performance in cohorts below 200 patients is prone to optimistic bias and that patient-level generalization is the relevant standard [19,20,21,22,23]; the cross-validation strategy applied here guards against the most common form of data leakage in repeated-measure datasets.

Domain-specific performance differences are biologically plausible: the combined score achieved the highest κ (0.152), consistent with averaging across domains reducing noise. Motor outcome was the notable exception, with a balanced accuracy of 0.277 falling below chance level (0.333); the classifier failed to achieve meaningful discrimination for this domain, likely reflecting the particularly severe class imbalance (74% in Group 3) combined with the limited sample size. Language showed the lowest κ (0.042) and the highest outcome variability (CV = 60%), reflecting its known sensitivity to post-discharge environmental determinants—parental language exposure, socioeconomic context, and early intervention access—that were not available in this dataset [23,29]. Comparison across all five algorithms (Supplementary Table S5) revealed no uniformly superior model: SVM-RBF performed best for cognitive and combined outcomes, while KNN showed competitive performance for motor and combined domains.

*Feature Importance and the MDI–Spearman Discrepancy:* Spearman r captures marginal, monotone associations in isolation; MDI quantifies each feature’s contribution within a multivariate ensemble [34,35]. This distinction explains why volume trajectory linearity (R^2^) ranked first in MDI for language and among the top three for motor and combined outcomes yet showed no significant univariate correlation—its predictive value is conditional rather than marginal, confirmed by permutation testing for motor outcome (*p* = 0.030). NIV duration, conversely, showed both high univariate Spearman r (r = −0.27 to −0.33, *p* < 0.05 across domains) and consistently high MDI—making it the most robustly validated predictor across all analytical levels. This multi-level convergence also holds for mechanical ventilation duration and BPD severity, reinforcing the extensive literature linking cumulative respiratory burden to impaired neurodevelopment through chronic hypoxia, neuroinflammatory cascades, and disrupted somatic and cerebral growth [4,5,6,7,38]. It bears emphasis that MDI-based importance and 2D partial dependence plots are derived from a Random Forest model trained on the full dataset, not from the primary SVM classifier. Random Forest and SVM operate under different inductive assumptions and may weight predictors differently; the feature importance findings should therefore be interpreted as a model-specific, exploratory characterization of the predictor–outcome landscape rather than an explanation of SVM decision boundaries.

*Clinical Implications:* The present study does not produce a clinically deployable prediction model, and results should not be used to guide individual clinical decisions at this stage. Sensitivity, specificity, and meaningful classification thresholds cannot be established from a single exploratory cohort without external validation.

The practical value of the findings lies at three levels. First, the robust association of respiratory morbidity duration—mechanical ventilation and BPD severity—with outcome across all analytical levels reinforces the clinical priority of minimizing ventilation exposure as a modifiable risk factor and may help direct preventive efforts toward infants with the highest cumulative respiratory burden. Second, the conditional contribution of serial CUS-derived brain volume trajectory features to multivariate classification suggests that longitudinal bedside volumetry may provide prognostic information complementary to standard clinical variables—particularly in the most immature infants—potentially supporting earlier identification of infants at risk and more targeted referral for developmental assessment and early intervention. Third, the identified interaction structures suggest that risk stratification algorithms should account for gestational maturity as an effect modifier rather than applying uniform thresholds across all GA groups [11,12,13,14,15,17,18]. Realizing this translational potential requires prospective multicenter validation, formal assessment of sensitivity and specificity in independent cohorts, and evaluation of whether CUS trajectory features improve clinical decision-making compared to standard care. Until then, findings should be regarded as hypothesis-generating and directionally informative.

*Limitations and Future Directions:* The cohort of 89 patients represents the primary limitation of this study as follows: with ~33 input features, the feature-to-sample ratio is unfavorable for ML, and confidence intervals around all performance estimates are wide. Patient-level GroupKFold cross-validation and a regularized SVM mitigate data leakage and overfitting, and the modest performance observed is itself inconsistent with gross overfitting—but external validation in an independent multicenter cohort remains an essential prerequisite before any clinical translation, and all results should be regarded as exploratory until then. Additionally, the absence of nested cross-validation means that model selection and performance estimation were conducted within the same cross-validation structure; the reported metrics for the selected SVM-RBF model may therefore carry optimistic bias and should not be interpreted as unbiased generalization estimates. Formal calibration analysis was not performed, and the identified interaction effects could in principle be investigated using conventional regression with pre-specified interaction terms; the ML framework used here offers the practical advantage of simultaneous data-driven screening across all predictor pairs without prior specification. Exclusion of severe brain injury limits applicability to the highest-risk infants. The ellipsoid ultrasound model provides a global whole-brain growth surrogate only; without concurrent MRI validation, the biological substrate of the reported trajectory features remains uncertain, and contributions from parenchymal growth, fluid redistribution, or measurement variability cannot be distinguished. Regional development, white matter microstructure, and network connectivity—all accessible by MRI—are not captured [1,8,30,31,32,33]. Post-discharge environmental determinants—socioeconomic factors, parental language environment, and early intervention access—were unavailable and represent a systematic residual variance source, particularly for language outcomes. Maternal gestational conditions—including autoimmune, endocrinological, neurological, psychiatric, and gynecological diseases, as well as maternal drug exposure during pregnancy—were not systematically collected and could not be included in the analyses. These factors may influence fetal neurodevelopment and cerebral growth trajectories The identified interaction effects are hypothesis-generating and require confirmatory testing in independent prospective cohorts.

Future work should prioritize multicenter prospective validation with pre-specified protocols, formal testing of the identified interaction hypotheses, and integration of nutritional and physiologic instability variables [39]. Fusion with MRI biomarkers at term-equivalent age would clarify the incremental value of bedside CUS volumetry [30,31,32,33]. Linking prediction outputs to actionable clinical pathways—early intervention thresholds, risk-stratified follow-up—will be required to demonstrate real-world benefit beyond statistical characterization [23].

## 5. Conclusions

This study suggests that a structured three-level machine learning framework—combining univariate inference, multivariate classification, and feature importance analysis with interaction characterization—may help characterize predictor–outcome relationships in preterm neurodevelopmental research, even where individual-level prediction remains out of reach. Respiratory morbidity duration—particularly NIV and mechanical ventilation—appeared as the most consistently associated predictor class across all analytical levels, while serial CUS-derived brain volume trajectory features showed conditional associations detectable only within a multivariate context. Interaction analyses indicated that the shape and consistency of the brain volume growth curve—rather than absolute volume alone—may carry context-dependent prognostic information, and that postnatal somatic growth restriction appeared to exert a more pronounced adverse effect in the most vulnerable infants. Given the exploratory nature of the analysis, the modest cohort size, and the absence of external validation, all findings should be regarded as hypothesis-generating. Multicenter validation in larger independent cohorts is required before any clinical translation.

## Figures and Tables

**Table 1:** Baseline characteristics of the study cohort (*n* = 89). Continuous variables are presented as mean ± SD or median (IQR). Abr.: APGAR = appearance, pulse, grimace, activity, respiration; GA = gestational age; SD = standard deviation; IMV = invasive mechanical ventilation; NIV = non-invasive ventilation; CS = cesarean section. *** *p* < 0.001 for birth vs. discharge (Wilcoxon signed-rank test).

Demographic Data *n* = 89	Birth		Discharge	
	Mean ± SD	Range/%	Mean ± SD	Range/%
GA (week + day)	28.3 ± 2.0	23.4–31.9		
Weight (g)	1019 ± 285	395–1490	2392 ± 381	1900–3970
Weight percentile	34 ± 24	1–94	12 ± 14 ***	1–68
Length (cm)	36.3 ± 3.7	27.0–43.0	44.5 ± 3.4	30.5–52.0
Length percentile	39 ± 22	2–81	9 ± 12 ***	1–67
Head circumference (cm)	25.7 ± 2.5	20.0–30.0	32.4 ± 2.9	28.5–48.5
Head circumference percentile	36 ± 24	2–86	12 ± 14 ***	1–68
APGAR 1	Median: 6	IQR: 5/7		
APGAR 5	Median: 8	IQR: 7/9		
APGAR 10	Median: 9	IQR: 8/9		
pH umbilical artery	Median: 7.33	IQR: 7.25/7.37		
IMV (h)	Median: 0	IQR: 0/72		
NIV (h)	Median: 744	IQR: 384/1128		
IMV (yes/no)	37/52	42/58%		
NIV (yes/no)	88/1	99/1%		
Sex (male/female)	36/53	40/60%		
Delivery (spontaneous/CS)	3/86	3/97%		

**Table 2:** Neonatal morbidities (*n* = 89). Data are presented as absolute numbers and percentages. Abr.: PDA = patent ductus arteriosus; BPD = bronchopulmonary dysplasia; AIS = amnion infection syndrome; EOS = early-onset sepsis; LOS = late-onset sepsis; BC = blood culture; NEC = necrotizing enterocolitis; FIP = focal intestinal perforation; IVH = intraventricular hemorrhage.

Morbidity Data	Overall *n* (%)	Category	*n* (%)
PDA	20 (22%)		
		None	69 (78%)
		Medical treatment	19 (21%)
		Surgical treatment	1 (1%)
BPD	13 (15%)		
		None	76 (85%)
		Mild	8 (9%)
		Moderate	3 (3%)
		Severe	2 (2%)
Sepsis	29 (33%)		
		None	60 (67%)
		AIS	3 (3%)
		EOS BC negative	1 (1%)
		EOS BC positive	3 (3%)
		LOS BC negative	9 (10%)
		LOS BC positive	13 (15%)
NEC/FIP	4 (4%)		
		None	85 (96%)
		Medical treatment	1 (1%)
		Surgical treatment	3 (3%)
IVH	12 (13%)	None	77 (87%)
		Grade I°	9 (10%)
		Grade II°	3 (3%)

**Table 3:** Brain volume measurements and growth parameters (*n* = 89). Brain volumes were assessed by 3D cranial ultrasound. Volumetric brain growth rate was calculated as absolute volume change from first to last measurement divided by the observation period in days. All values are presented as mean ± SD with range.

Brain Volume Measurements (*n* = 89)	Mean ± SD	Range
Brain volume 1st measurement (cm^3^)	158 ± 38	77–243
Brain volume last measurement (cm^3^)	275 ± 56	140–454
Volumetric brain growth rate (cm^3^/day)	2.4 ± 0.9	−0.84–5.39
Number of ultrasound examinations per infant	6.6 ± 2.3	2–13

**Table 4:** Bayley-III outcomes at 24 months corrected age (*n* = 89). All domains are reported as percentile ranks (normative mean = 50th percentile). Values are presented as mean ± SD with range.

Bayley-III Domain (Percentile Rank)	Mean ± SD	Range
Cognitive	72 ± 31	1–100
Language	55 ± 33	1–98
Motor	67 ± 25	2–99
Combined	65 ± 26	1–99

**Table 5:** Performance of SVM classifier with RBF kernel (*n* = 89, 5-fold GroupKFold CV). Abr.: SD = standard deviation; F1 = F1-score (macro-averaged); κ = Cohen’s kappa (quadratic weighted); MAE = mean absolute error; RMSE = root mean squared error; R^2^ = coefficient of determination (class units, scale 1–3). κ > 0.2 = fair agreement; κ > 0.4 = moderate agreement.

Bayley-III Domain	Balanced Accuracy (Mean ± SD)	F1-Score (Macro)	Cohen’s κ (Quadr. wt.)	MAE (Class Units)	RMSE (Class Units)	R^2^
Cognitive	0.377 (±0.103)	0.374	0.128	0.640	1.033	−1.451
Language	0.331 (±0.106)	0.308	0.042	0.910	1.250	−1.429
Motor	0.277 (±0.079)	0.308	0.075	0.674	1.060	−2.170
Combined	0.463 (±0.135)	0.349	0.152	0.775	1.137	−2.334

## Data Availability

Clinical data were recorded and stored in pseudonymized form within the study-site network for 10 years (§630f BGB). No data are shared with third parties; access is restricted to the study team via the hospital network and a password-protected database. Public reporting uses anonymized data only. De-identified datasets generated and/or analyzed during the study are available from the corresponding author on reasonable request.

## Supplementary Materials

The following supporting information can be downloaded at: https://www.mdpi.com/article/10.3390/children13060815/s1, Figure S1. Spearman rank correlation matrix of all predictor variables; Figure S2. Two-dimensional partial dependence plots of the top-4 feature interactions for Bayley-III Cognitive outcome; Figure S3. Two-dimensional partial dependence plots of the top-4 feature interactions for Bayley-III Language outcome; Figure S4. Two-dimensional partial dependence plots of the top-4 feature interactions for Bayley-III Motor outcome; Figure S5. Two-dimensional partial dependence plots of the top-4 feature interactions for Bayley-III Combined outcome; Table S1. Top-10 feature interactions for Bayley-III Cognitive outcome ranked by stratified Spearman Δr; Table S2. Top-10 feature interactions for Bayley-III Language outcome ranked by stratified Spearman; Table S3. Top-10 feature interactions for Bayley-III Motor outcome ranked by stratified Spearman Δr; Table S4. Top-10 feature interactions for Bayley-III Combined outcome ranked by stratified Spearman Δr; Table S5. Cross-validated performance of all five classification algorithms across four Bayley-III outcome domains.
