# Supplementary material for: Neurodevelopmental Outcome in Very Low Birth Weight Preterm Infants: An Exploratory Multivariable Analysis Including Sonographic Brain Volume Trajectories—Data from the NeoNEVS Project
Source: Children (Basel). 2026 Jun 13;13(6):815. doi: 10.3390/children13060815 (PMC13297280; doi:10.3390/children13060815)
Supplement: Supplementary file 1 [file children-13-00815-s001.zip › children-4340991-supplementary.pdf]

Supplemental material:

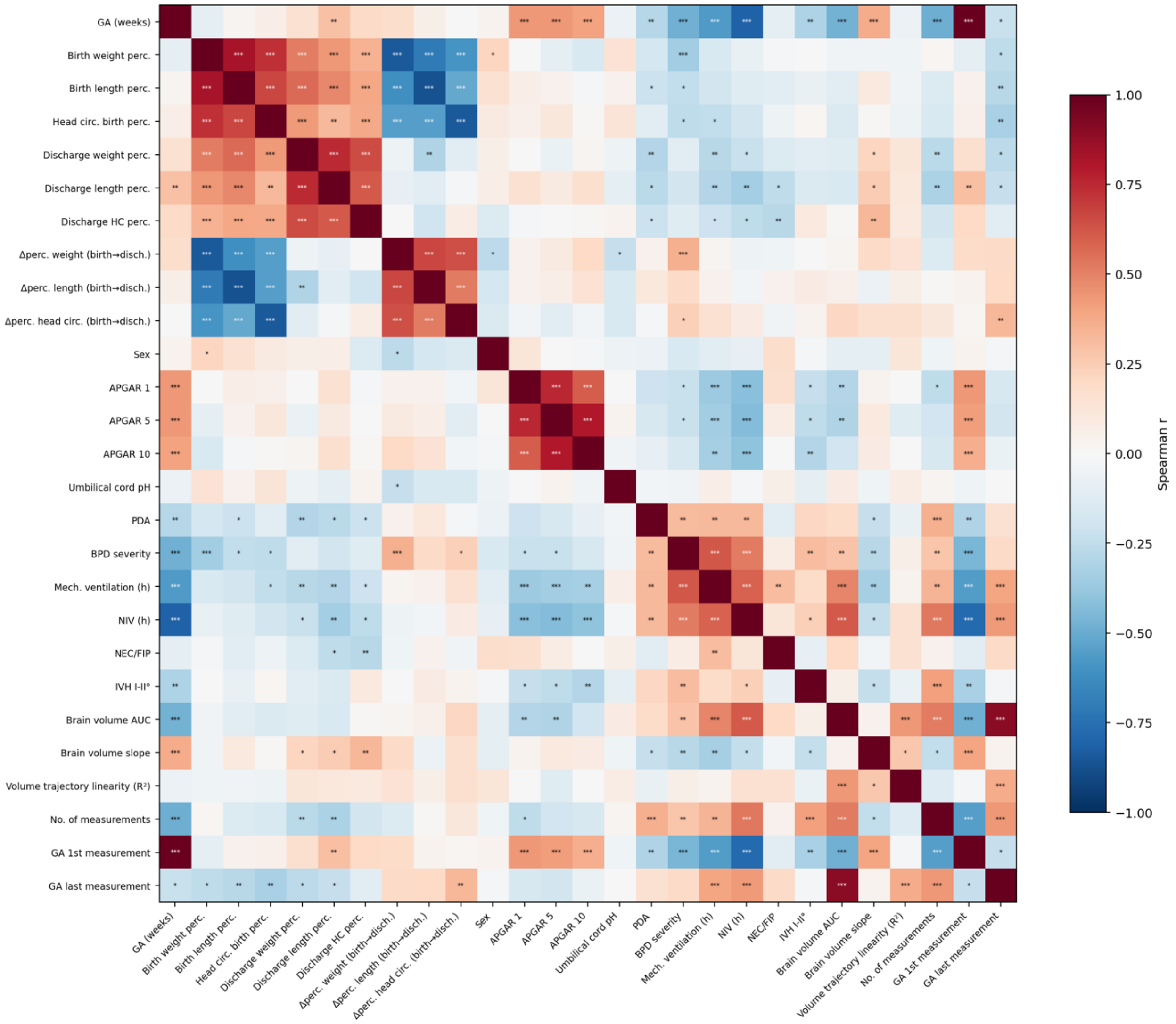

Supplementary Figure S1. *Spearman rank correlation matrix of all predictor variables (n=89). Color encodes the Spearman rank correlation coefficient between each pair of predictors: red = positive correlation, blue = negative correlation, white = no correlation. Asterisks indicate statistical significance (* p<0.05; ** p<0.01; *** p<0.001). Notable collinearities include the intercorrelations between BPD severity, mechanical ventilation duration, and NIV duration (r = 0.52–0.62), reflecting the biological interdependence of respiratory morbidity markers, and the strong correlation between GA at birth and NIV duration (r = −0.81), reflecting the well-established inverse relationship between gestational maturity and respiratory support requirement. Brain volume AUC and GA at last measurement showed high collinearity (r = +0.90), consistent with the dependence of cumulative volume on observation duration. All variables were retained for the multivariate analysis; collinearity is acknowledged as a potential source of redundancy in predictor contributions but does not affect the validity of SVM or Random Forest classifiers, which are robust to correlated input features. Abr.: GA = Gestational Age; BPD = Bronchopulmonary Dysplasia; NIV = Non-Invasive Ventilation; AUC = Area Under the Curve; HC = Head Circumference; Δperc. = postnatal percentile change birth to discharge.*

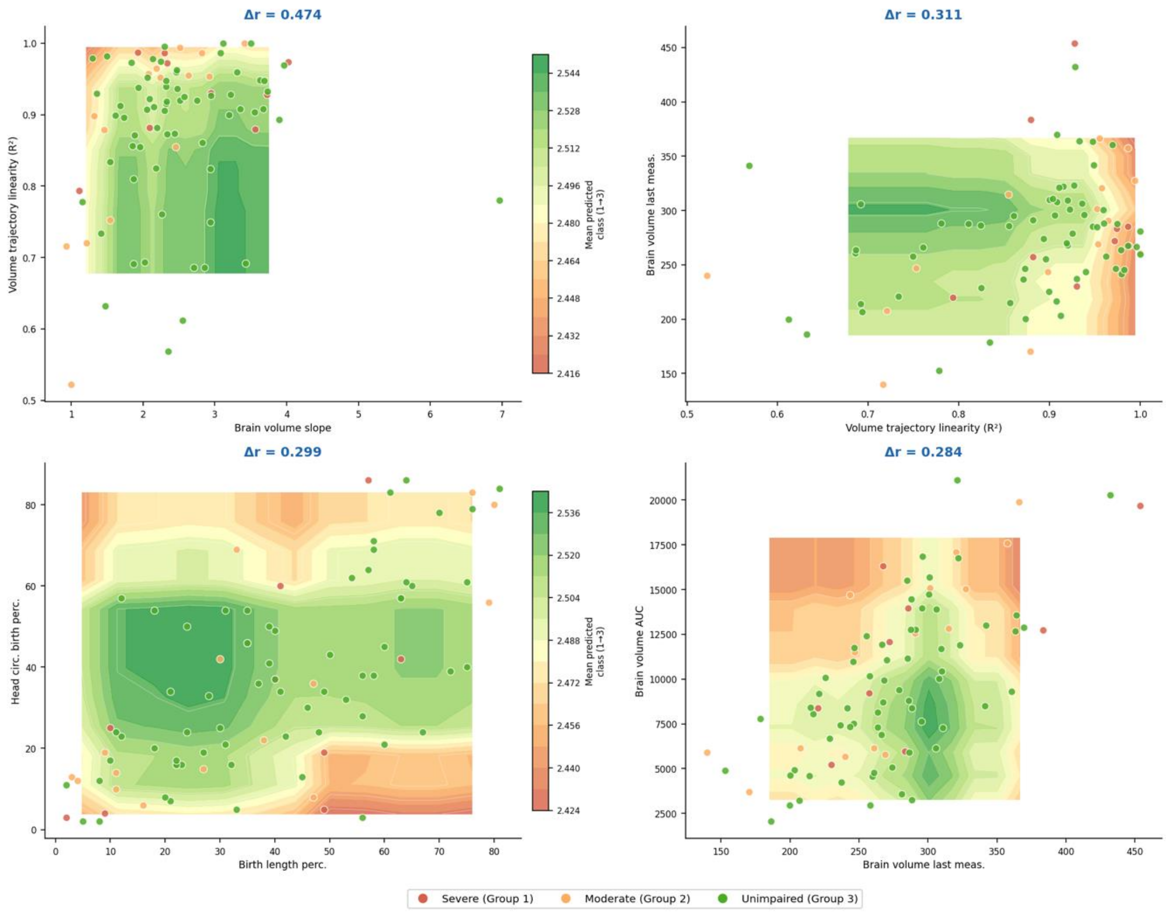

Supplementary Figure S2. *Two-dimensional partial dependence plots of the top-4 feature interactions for Bayley-III Cognitive outcome (n=89). For each feature pair, the background color encodes the mean predicted outcome class averaged across cross-validation folds, estimated by a Random Forest classifier (n=50 trees) and computed over a 12×12 grid spanning the observed feature range: green indicates higher mean predicted class (better outcome, Group 3), red indicates lower mean predicted class (worse outcome, Group 1). Dots represent individual patients, colored by their true outcome group (red = Group 1: severely impaired, percentile rank ≤16; orange = Group 2: moderately impaired, percentile rank 17–50; green = Group 3: unimpaired, percentile rank >50). Feature pairs are ranked by interaction strength Δr, defined as the absolute difference in stratified Spearman correlations between subgroups split at the median of the stratification variable. All results are exploratory and hypothesis-generating. Abr.: Δr = stratified Spearman interaction strength metric; R^2 = volume trajectory linearity; AUC = Area Under the Curve; Δperc. = postnatal percentile change (birth to discharge).*

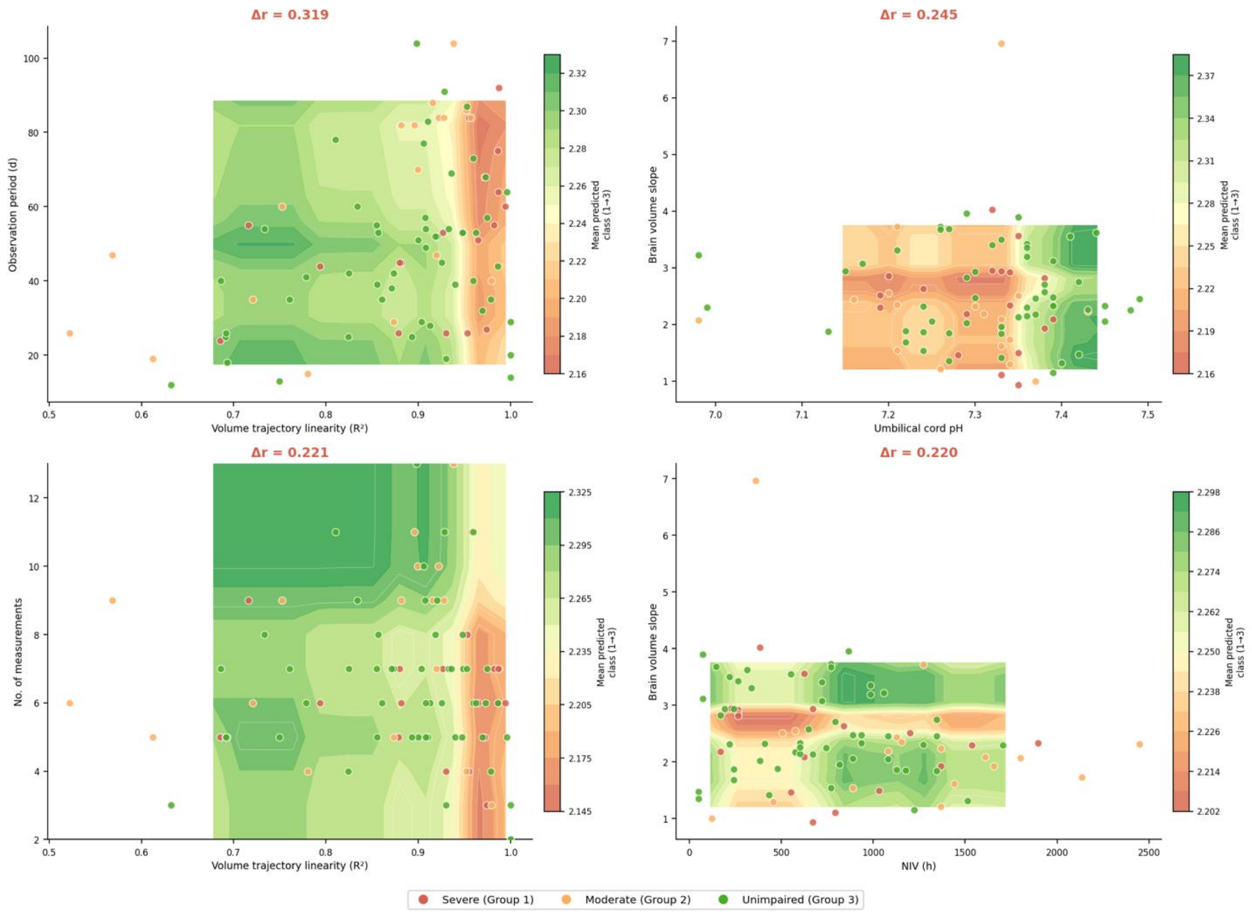

Supplementary Figure S3. *Two-dimensional partial dependence plots of the top-4 feature interactions for Bayley-III Language outcome (n=89). For each feature pair, the background color encodes the mean predicted outcome class averaged across cross-validation folds, estimated by a Random Forest classifier (n=50 trees) and computed over a 12×12 grid spanning the observed feature range: green indicates higher mean predicted class (better outcome, Group 3), red indicates lower mean predicted class (worse outcome, Group 1). Dots represent individual patients, colored by their true outcome group (red = Group 1: severely impaired, percentile rank ≤16; orange = Group 2: moderately impaired, percentile rank 17–50; green = Group 3: unimpaired, percentile rank >50). Feature pairs are ranked by interaction strength Δr, defined as the absolute difference in stratified Spearman correlations between subgroups split at the median of the stratification variable. All results are exploratory and hypothesis-generating. Abr.: Δr = stratified Spearman interaction strength metric; R^2 = volume trajectory linearity; AUC = Area Under the Curve; Δperc. = postnatal percentile change (birth to discharge).*

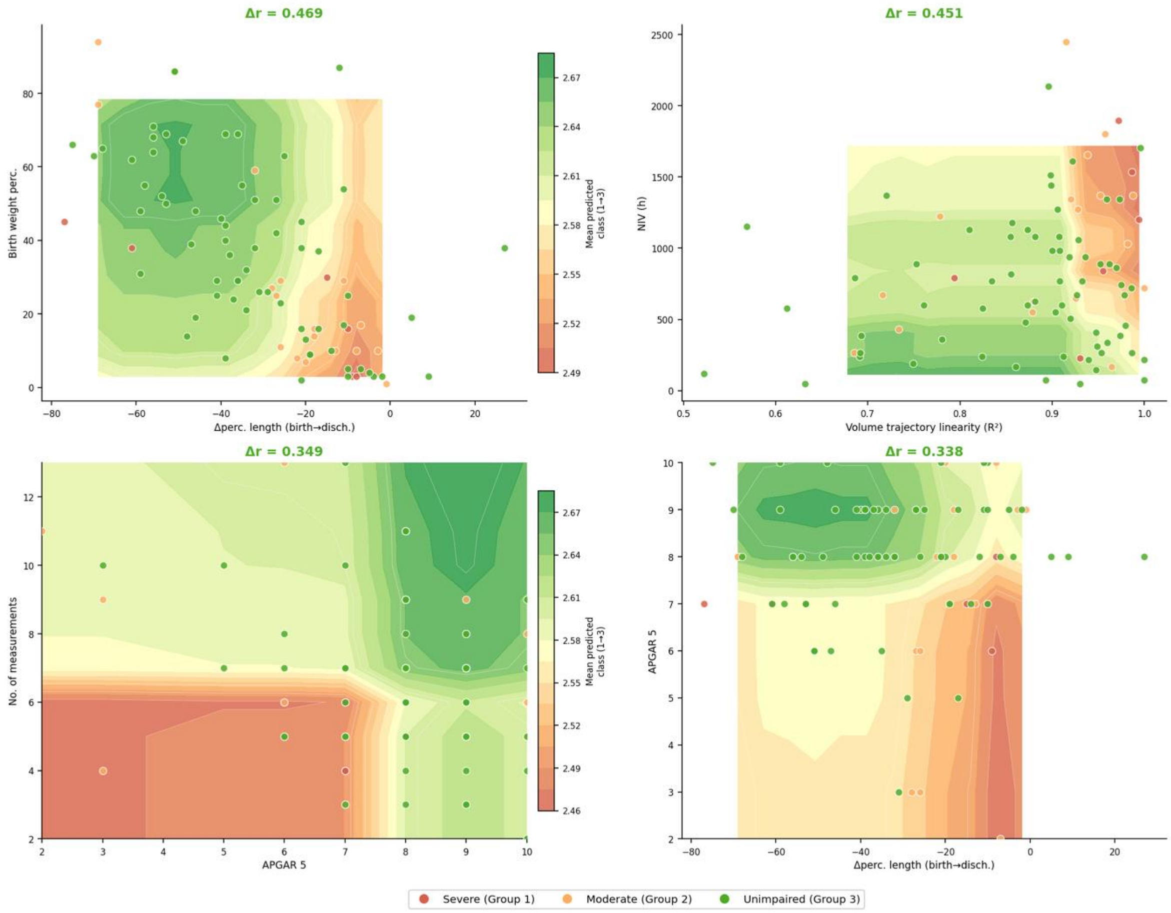

Supplementary Figure S4. *Two-dimensional partial dependence plots of the top-4 feature interactions for Bayley-III Motor outcome (n=89). For each feature pair, the background color encodes the mean predicted outcome class averaged across cross-validation folds, estimated by a Random Forest classifier (n=50 trees) and computed over a 12×12 grid spanning the observed feature range: green indicates higher mean predicted class (better outcome, Group 3), red indicates lower mean predicted class (worse outcome, Group 1). Dots represent individual patients, colored by their true outcome group (red = Group 1: severely impaired, percentile rank ≤16; orange = Group 2: moderately impaired, percentile rank 17–50; green = Group 3: unimpaired, percentile rank >50). Feature pairs are ranked by interaction strength Δr, defined as the absolute difference in stratified Spearman correlations between subgroups split at the median of the stratification variable. All results are exploratory and hypothesis-generating. Abr.: Δr = stratified Spearman interaction strength metric; R^2 = volume trajectory linearity; AUC = Area Under the Curve; Δperc. = postnatal percentile change (birth to discharge).*

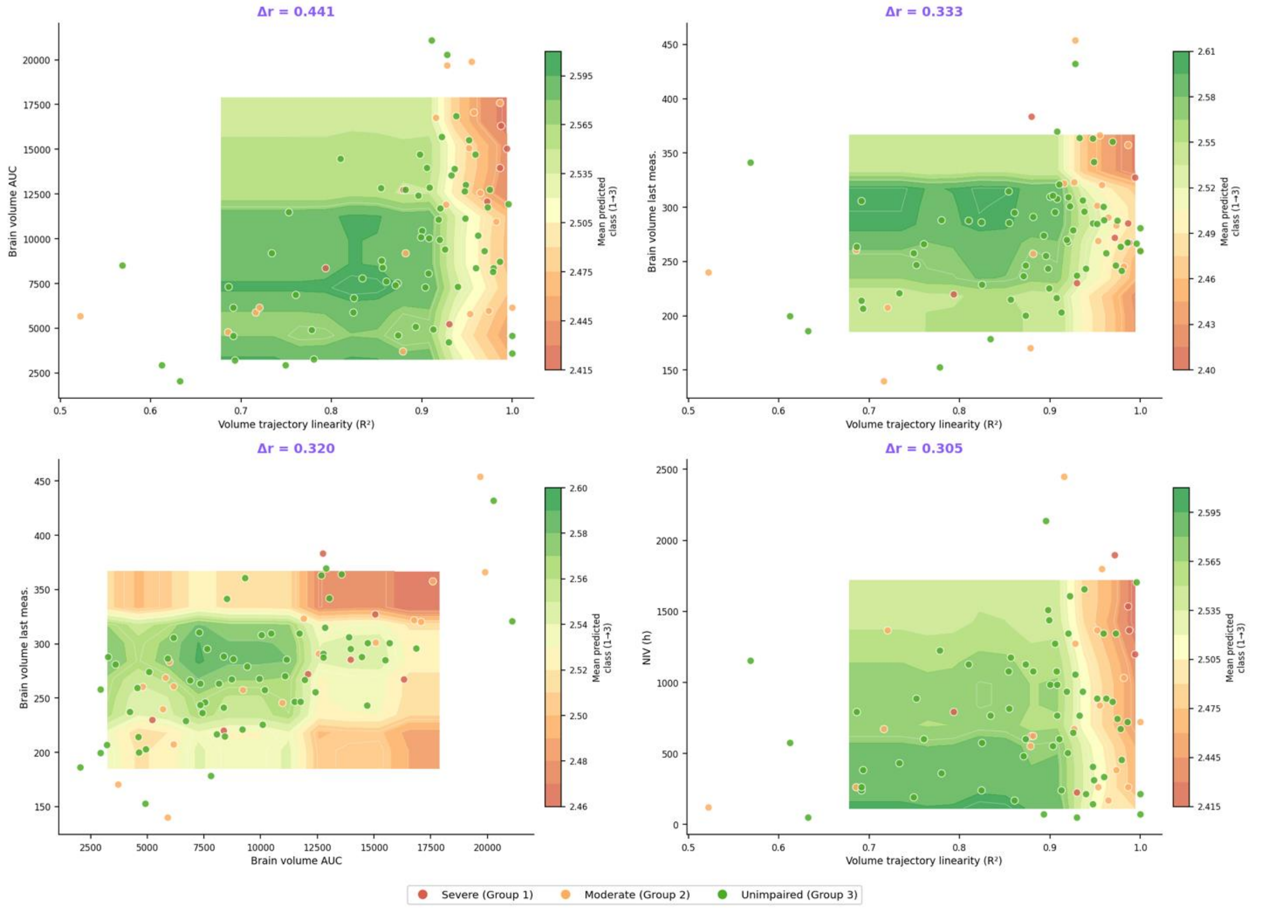

Supplementary Figure S5. *Two-dimensional partial dependence plots of the top-4 feature interactions for Bayley-III Combined outcome (n=89). For each feature pair, the background color encodes the mean predicted outcome class averaged across cross-validation folds, estimated by a Random Forest classifier (n=50 trees) and computed over a 12×12 grid spanning the observed feature range: green indicates higher mean predicted class (better outcome, Group 3), red indicates lower mean predicted class (worse outcome, Group 1). Dots represent individual patients, colored by their true outcome group (red = Group 1: severely impaired, percentile rank ≤16; orange = Group 2: moderately impaired, percentile rank 17–50; green = Group 3: unimpaired, percentile rank >50). Feature pairs are ranked by interaction strength Δr, defined as the absolute difference in stratified Spearman correlations between subgroups split at the median of the stratification variable. All results are exploratory and hypothesis-generating. Abr.: Δr = stratified Spearman interaction strength metric; R² = volume trajectory linearity; AUC = Area Under the Curve; Δperc. = postnatal percentile change (birth to discharge).*

Predictor (X)	Stratification variable (Z)	n (low Z)	r(X→outcome) when Z low	n (high Z)	r(X→outcome) when Z high	Δr (interaction strength)
Brain volume slope	Volume trajectory linearity (R^2)	n=45	+0.380 *	n=44	-0.095	**0.474**
Volume trajectory linearity (R^2)	Brain volume last meas.	n=45	-0.049	n=44	-0.359 *	**0.311**
Birth length perc.	Head circ. birth perc.	n=47	+0.255	n=42	-0.044	**0.299**
Brain volume last meas.	Brain volume AUC	n=45	+0.165	n=44	-0.120	0.284
Birth weight perc.	Volume trajectory linearity (R^2)	n=45	+0.047	n=44	+0.328 *	0.281
Brain volume slope	Birth length perc.	n=45	+0.210	n=44	-0.048	0.258
Birth length perc.	Brain volume last meas.	n=45	+0.243	n=44	-0.006	0.249
Birth length perc.	Volume trajectory linearity (R^2)	n=45	+0.016	n=44	+0.255	0.239
Head circ. birth perc.	Volume trajectory linearity (R^2)	n=45	+0.017	n=44	+0.251	0.234
Brain volume slope	Birth weight perc.	n=46	+0.172	n=43	-0.048	0.219

*Δr = |r(X→outcome | Z high) − r(X→outcome | Z low)| | Stratification at median of Z | * p<0.05 ** p<0.01 *** p<0.001*

Supplementary Table S1. *Top-10 feature interactions for Bayley-III Cognitive outcome ranked by stratified Spearman Δr (n=89, corrected cutoffs). For each feature pair, Predictor X and Stratification Variable Z are listed alongside the Spearman rank correlation between X and the Cognitive outcome group assignment in the low-Z stratum (patients with Z ≤ median of Z) and the high-Z stratum (patients with Z > median of Z), including subgroup sample sizes (n). Outcome groups are defined by Bayley-III percentile rank: Group 1 = severely impaired (≤16th percentile), Group 2 = moderately impaired (17th–50th percentile), Group 3 = unimpaired (>50th percentile). Δr denotes the absolute difference between the two stratum-specific Spearman correlations and serves as the primary measure of interaction strength: a high Δr indicates that the predictive association of X with outcome changes substantially depending on the level of Z. P-values for stratum-specific correlations are annotated (* p<0.05; ** p<0.01; *** p<0.001). Yellow shading highlights the three feature pairs with the highest interaction strength. All results are exploratory and hypothesis-generating; formal confirmatory testing in an independent prospective cohort is required. Abr.: Δr = stratified Spearman interaction strength metric; AUC = Area Under the Curve; R^2 = volume trajectory linearity; Δperc. = postnatal percentile change birth to discharge.*

Predictor (X)	Stratification variable (Z)	n (low Z)	r(X→outcome) when Z low	n (high Z)	r(X→outcome) when Z high	Δr (interaction strength)
Volume trajectory linearity (R^2)	Observation period (d)	n=46	+0.057	n=43	-0.263	**0.319**
Umbilical cord pH	Brain volume slope	n=45	+0.019	n=44	+0.264	**0.245**
Volume trajectory linearity (R^2)	No. of measurements	n=47	+0.007	n=42	-0.214	**0.221**
NIV (h)	Brain volume slope	n=45	-0.259	n=44	-0.040	0.220
Volume trajectory linearity (R^2)	NIV (h)	n=45	+0.011	n=44	-0.204	0.215
No. of measurements	GA 1st measurement	n=45	+0.118	n=44	-0.092	0.209
Umbilical cord pH	APGAR 1	n=50	+0.259	n=39	+0.053	0.206
NIV (h)	Observation period (d)	n=46	-0.053	n=43	-0.258	0.205
NIV (h)	GA 1st measurement	n=45	-0.199	n=44	+0.003	0.202
No. of measurements	Observation period (d)	n=46	-0.106	n=43	+0.094	0.200

*Δr = |r(X→outcome | Z high) − r(X→outcome | Z low)| | Stratification at median of Z | * p<0.05 ** p<0.01 *** p<0.001*

Supplementary Table S2. *Top-10 feature interactions for Bayley-III Language outcome ranked by stratified Spearman Δr (n=89, corrected cutoffs). For each feature pair, Predictor X and Stratification Variable Z are listed alongside the Spearman rank correlation between X and the Cognitive outcome group assignment in the low-Z stratum (patients with Z ≤ median of Z) and the high-Z stratum (patients with Z > median of Z), including subgroup sample sizes (n). Outcome groups are defined by Bayley-III percentile rank: Group 1 = severely impaired (≤16th percentile), Group 2 = moderately impaired (17th–50th percentile), Group 3 = unimpaired (>50th percentile). Δr denotes the absolute difference between the two stratum-specific Spearman correlations and serves as the primary measure of interaction strength: a high Δr indicates that the predictive association of X with outcome changes substantially depending on the level of Z. P-values for stratum-specific correlations are annotated (* p<0.05; ** p<0.01; *** p<0.001). Yellow shading highlights the three feature pairs with the highest interaction strength. All results are exploratory and hypothesis-generating; formal confirmatory testing in an independent prospective cohort is required. Abr.: Δr = stratified Spearman interaction strength metric; AUC = Area Under the Curve; R^2 = volume trajectory linearity; Δperc. = postnatal percentile change birth to discharge.*

Predictor (X)	Stratification variable (Z)	n (low Z)	r(X→outcome) when Z low	n (high Z)	r(X→outcome) when Z high	Δr (interaction strength)
Δperc. length (birth→disch.)	Birth weight perc.	n=46	-0.243	n=43	+0.227	**0.469**
Volume trajectory linearity (R²)	NIV (h)	n=45	+0.057	n=44	-0.393 **	**0.451**
APGAR 5	No. of measurements	n=47	+0.335 *	n=42	-0.014	**0.349**
Δperc. length (birth→disch.)	APGAR 5	n=57	-0.108	n=32	-0.447 *	0.338
Δperc. length (birth→disch.)	NIV (h)	n=45	-0.372 *	n=44	-0.085	0.287
NIV (h)	Birth weight perc.	n=46	-0.358 *	n=43	-0.075	0.284
APGAR 5	NIV (h)	n=45	-0.017	n=44	+0.241	0.259
Birth weight perc.	Brain volume slope	n=45	+0.337 *	n=44	+0.100	0.237
Δperc. length (birth→disch.)	Volume trajectory linearity (R²)	n=45	-0.097	n=44	-0.333 *	0.236
Volume trajectory linearity (R²)	APGAR 5	n=57	-0.245	n=32	-0.037	0.208

*Δr = |r(X→outcome | Z high) − r(X→outcome | Z low)| | Stratification at median of Z | * p<0.05 ** p<0.01 *** p<0.001*

Supplementary Table S3. *Top-10 feature interactions for Bayley-III Motor outcome ranked by stratified Spearman Δr (n=89, corrected cutoffs). For each feature pair, Predictor X and Stratification Variable Z are listed alongside the Spearman rank correlation between X and the Cognitive outcome group assignment in the low-Z stratum (patients with Z ≤ median of Z) and the high-Z stratum (patients with Z > median of Z), including subgroup sample sizes (n). Outcome groups are defined by Bayley-III percentile rank: Group 1 = severely impaired (≤16th percentile), Group 2 = moderately impaired (17th–50th percentile), Group 3 = unimpaired (>50th percentile). Δr denotes the absolute difference between the two stratum-specific Spearman correlations and serves as the primary measure of interaction strength: a high Δr indicates that the predictive association of X with outcome changes substantially depending on the level of Z. P-values for stratum-specific correlations are annotated (* p<0.05; ** p<0.01; *** p<0.001). Yellow shading highlights the three feature pairs with the highest interaction strength. All results are exploratory and hypothesis-generating; formal confirmatory testing in an independent prospective cohort is required. Abr.: Δr = stratified Spearman interaction strength metric; AUC = Area Under the Curve; R² = volume trajectory linearity; Δperc. = postnatal percentile change birth to discharge.*

Predictor (X)	Stratification variable (Z)	n (low Z)	r(X→outcome) when Z low	n (high Z)	r(X→outcome) when Z high	Δr (interaction strength)
Volume trajectory linearity (R^2)	Brain volume AUC	n=45	+0.003	n=44	-0.438 **	**0.441**
Volume trajectory linearity (R^2)	Brain volume last meas.	n=45	-0.073	n=44	-0.406 **	**0.333**
Brain volume AUC	Brain volume last meas.	n=45	-0.023	n=44	-0.343 *	**0.320**
Volume trajectory linearity (R^2)	NIV (h)	n=45	-0.070	n=44	-0.375 *	0.305
Δperc. weight (birth→disch.)	Birth weight perc.	n=46	-0.096	n=43	-0.359 *	0.263
Brain volume AUC	NIV (h)	n=45	-0.067	n=44	-0.297	0.230
Δperc. weight (birth→disch.)	Brain volume AUC	n=45	-0.160	n=44	-0.376 *	0.216
Birth length perc.	Δperc. length (birth→disch.)	n=45	-0.027	n=44	+0.180	0.207
Volume trajectory linearity (R^2)	Δperc. length (birth→disch.)	n=45	-0.135	n=44	-0.305 *	0.170
NIV (h)	Brain volume last meas.	n=45	-0.048	n=44	-0.193	0.145

*Δr = |r(X→outcome | Z high) − r(X→outcome | Z low)| | Stratification at median of Z | * p<0.05 ** p<0.01 *** p<0.001*

Supplementary Table S4. *Top-10 feature interactions for Bayley-III Combined outcome ranked by stratified Spearman Δr (n=89, corrected cutoffs). For each feature pair, Predictor X and Stratification Variable Z are listed alongside the Spearman rank correlation between X and the Cognitive outcome group assignment in the low-Z stratum (patients with Z ≤ median of Z) and the high-Z stratum (patients with Z > median of Z), including subgroup sample sizes (n). Outcome groups are defined by Bayley-III percentile rank: Group 1 = severely impaired (≤16th percentile), Group 2 = moderately impaired (17th–50th percentile), Group 3 = unimpaired (>50th percentile). Δr denotes the absolute difference between the two stratum-specific Spearman correlations and serves as the primary measure of interaction strength: a high Δr indicates that the predictive association of X with outcome changes substantially depending on the level of Z. P-values for stratum-specific correlations are annotated (* p<0.05; ** p<0.01; *** p<0.001). Yellow shading highlights the three feature pairs with the highest interaction strength. All results are exploratory and hypothesis-generating; formal confirmatory testing in an independent prospective cohort is required. Abr.: Δr = stratified Spearman interaction strength metric; AUC = Area Under the Curve; R^2 = volume trajectory linearity; Δperc. = postnatal percentile change birth to discharge.*

Algorithm	Cognitive BalAcc	Cognitive κ	Language BalAcc	Language κ	Motor BalAcc	Motor κ	Combined BalAcc	Combined κ
Logistic Reg. (L2)	0.345	0.007	0.349	**0.055**	0.320	**0.146**	0.328	0.122
Naive Bayes	0.335	0.054	0.332	0.048	0.370	0.118	0.306	0.051
KNN (k=5)	**0.393**	**0.137**	0.350	-0.064	**0.392**	0.046	0.427	**0.208**
SVM (RBF)	0.377	0.128	0.331	0.042	0.277	0.075	**0.463**	0.152
Random Forest	0.333	0.064	**0.352**	0.008	0.343	0.044	0.328	-0.016

Supplementary Table S5. *Cross-validated performance of all five classification algorithms across four Bayley-III outcome domains (5-fold GroupKFold, patient-level grouping, n=89). Balanced Accuracy (mean across folds) and Cohen's κ (quadratic weighted) are reported for each algorithm–domain combination. Bold values indicate the best performance per column. Green shading identifies the SVM with RBF kernel, selected as the primary model. All algorithms used scikit-learn default hyperparameters with balanced class weighting: SVM-RBF (C=1, gamma='scale'); Logistic Regression (C=1, L2 regularization, max_iter=1000); Naive Bayes (GaussianNB, default priors); KNN (k=5, uniform weights); Random Forest (n_estimators=100, max_features='sqrt', no depth constraint). No hyperparameter tuning or nested cross-validation was applied; model selection was based on balanced accuracy on the same 5-fold structure, which is acknowledged as a potential source of mild optimistic bias. Abr.: BalAcc = Balanced Accuracy; κ = Cohen's Kappa (quadratic weighted); SVM = Support Vector Machine.*
